# Aortitis after administration of pegfilgrastim to a healthy donor for peripheral blood stem cell collection

**DOI:** 10.1007/s12185-023-03649-0

**Published:** 2023-08-14

**Authors:** Yu Uemura, Kumi Oshima, Aika Fuseya, Akane Hosokai, Ayaka Ohashi, Masatoshi Kanno, Ayako Arai

**Affiliations:** 1 Department of Hematology and Oncology, St. Marianna University School of Medicine, 2-16-1 Sugao, Miyamae-Ku, Kawasaki, 216-8511 Japan; 2 Department of Immunology and Parasitology, St. Marianna University School of Medicine, Kawasaki, Japan

**Keywords:** Pegfilgrastim, Aortitis, Donor, Allogeneic peripheral blood stem cell transplantation

## Abstract

A 45-year-old man who was a sibling donor for allogeneic peripheral blood stem cell transplantation (allo-PBSCT) was administered 7.2 mg of pegfilgrastim for stem cell collection. Peripheral blood stem cells were collected 4 days after administration of pegfilgrastim (Day 4) and 4.32 × 10^6^ /kg of CD34-positive cells per recipient body weight were obtained. Fever of 38 ℃ or higher and left submandibular pain appeared on Day 6. Ultrasonography and contrast-enhanced computed tomography (CT) showed wall thickening of the carotid artery and the abdominal aorta. We carefully excluded the possibilities of cardiovascular and autoimmune diseases by thorough examination, and ultimately diagnosed pegfilgrastim-induced aortitis. The patient’s fever resolved rapidly after treatment with prednisolone (PSL) 1 mg/kg. We began to taper PSL after eight days. Sixty-one days after starting PSL, we confirmed that abdominal aortic wall thickening had improved by contrast-enhanced CT. We continued to taper off PSL and stopped 141 days later with no relapse thereafter. This is the first case report of pegfilgrastim-induced aortitis in an allo-PBSCT donor. Careful monitoring is warranted when administering pegfilgrastim to donors even without past medical history.

## Introduction

Pegfilgrastim, a long-acting granulocyte colony-stimulating factor (G-CSF) preparation, has been widely used as a primary prophylaxis to prevent chemotherapy-associated febrile neutropenia. In recent years, the application has expanded to allogeneic peripheral blood stem cell collection, and this application is covered by the Japanese public health insurance since February 2022. When collecting hematopoietic stem cells with pegfilgrastim, donors’ hospitalization period and physical burden can be reduced. However, due to the limited experience of its use, there may be risks of unpredictable serious adverse events.

Aortitis is well known to occur in autoimmune diseases, but it has been reported also to occur during the use of drugs such as G-CSF [1]. Most reports of G-CSF-induced aortitis are of cancer patients [2, 3], and there is no report of aortitis developed in a healthy donor of hematopoietic stem cell transplantation (HSCT) after administering pegfilgrastim. Here we present the first report of such case of aortitis.

## Case

**Fig. 1:**
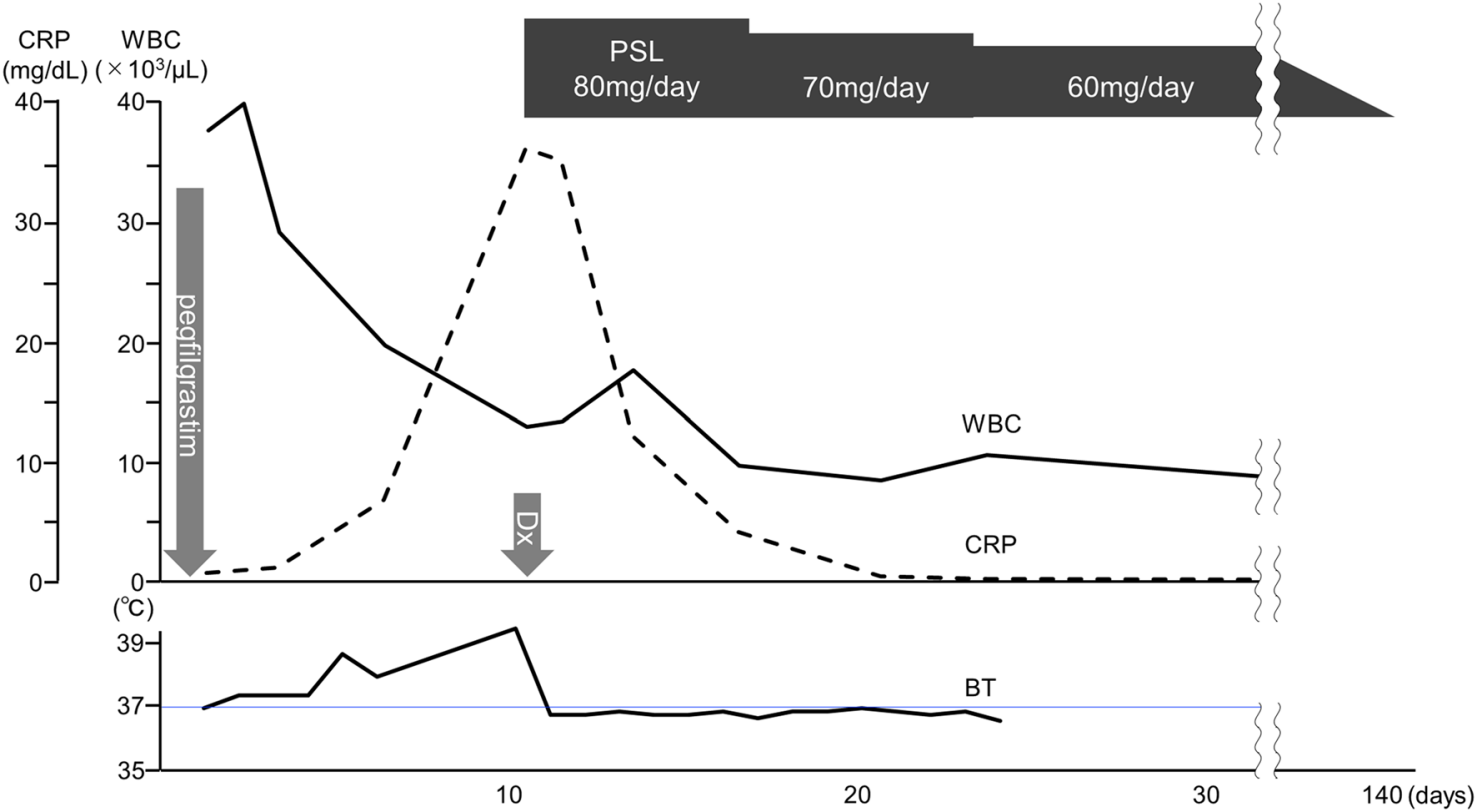
Clinical course of the patient. PSL; prednisolone, WBC; white blood cells, CRP; C-reactive protein, Dx; diagnosis, BT; body temperature

**Fig. 2:**
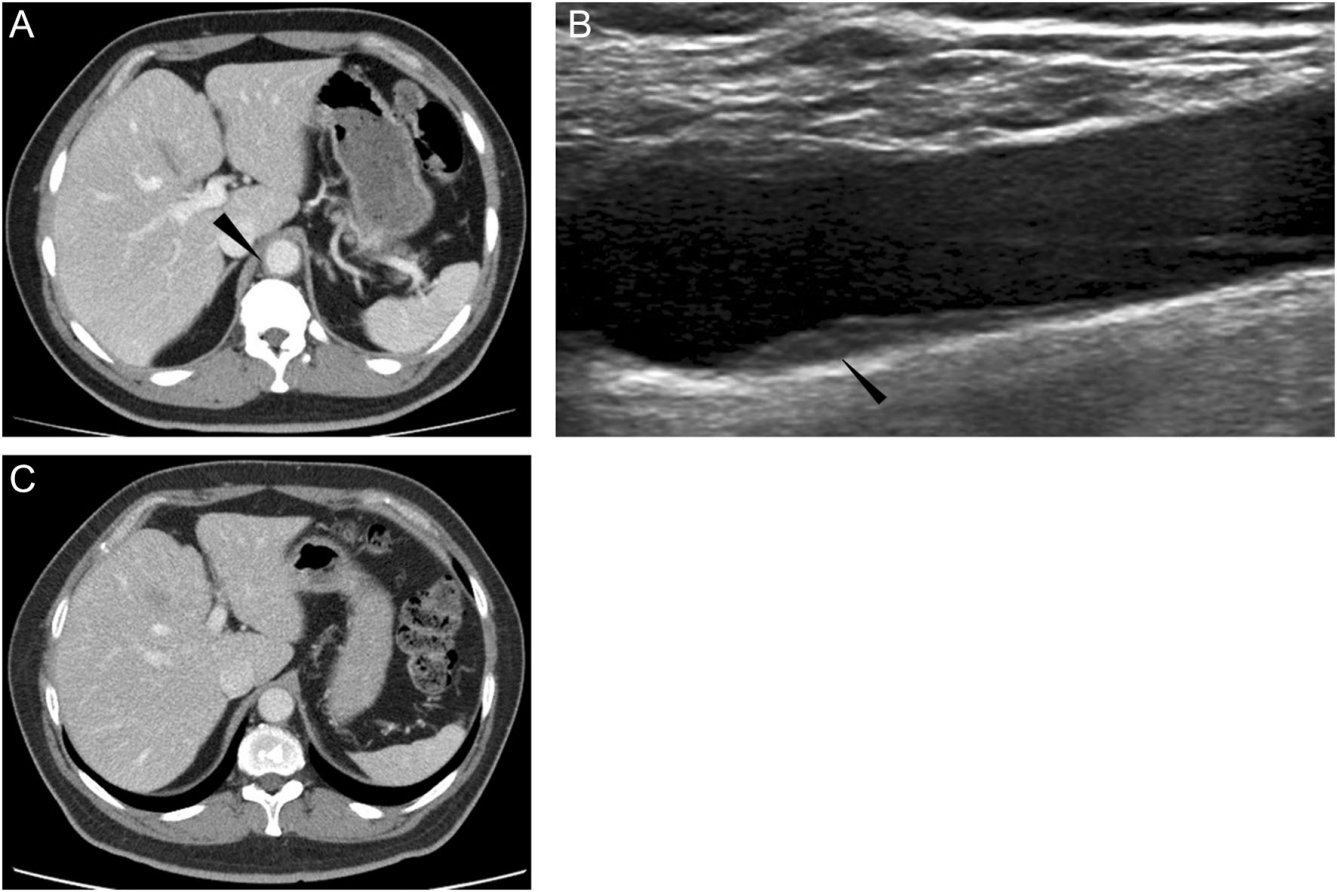
The image findings of the vascular lesions. A: abdominal contrast-enhanced computed tomography (CT) imaging at diagnosis. The aortic wall is thickened (arrow). B: carotid ultrasonography before treatment. The carotid artery thickened (arrow). C: abdominal contrast-enhanced CT imaging after treatment. Aortic wall thickening improved

The clinical course of a 45-year-old male is shown in Figure 1. He is the brother of a 37-year-old male patient of systemic chronic active Epstein–Barr disease (sCAEBV) and is the allogenic HSCT donor of his brother. His medical history included clavicle fracture and colonic diverticulitis. He received 7.2 mg of pegfilgrastim to collect peripheral blood stem cells. We set this date as Day 0 of our observation. On Day 4, 4.32×10^6^ /kg of CD34-positive peripheral blood stem cells were collected per recipient’s body weight. On Day 6, fever of 38 ℃ or higher was observed. On Day 8, he visited a local outpatient clinic because he started to feel left submandibular pain. During physical examination, mild tenderness was noted in his left mandibular angle. He was tested for SARS-CoV-2 polymerase chain reaction, and the result was negative. The physician in charge assumed that he had an infectious lymphadenitis and prescribed 500 mg/day levofloxacin and 500 mg of acetaminophen. However, his fever and left submandibular pain did not improve even on Day 12. He was hospitalized to investigate the cause of the fever. Contrast-enhanced computed tomography (CT) did not show any focus of infection but revealed the thickening of the abdominal aortic wall (Fig. 2A). The results of blood test at the time of his admission are shown on Table 1. The results presented an increase of neutrophils and elevated levels of C-reactive protein (CRP), fibrinogen, and ferritin (Table 1). We examined him by ultrasonography on Day 13 and confirmed the thickening of the carotid artery wall (Fig. 2B). Because he had no history nor evidence of cardiovascular disease, autoimmune disease, and presumed cause other than pegfilgrastim, we finally diagnosed him as pegfilgrastim-induced aortitis based on the clinical course. Just after we started the administration of 1 mg/kg/day prednisolone (PSL), his fever rapidly subsided and the value of CRP decreased. PSL was tapered to 60 mg/day and then he was discharged from the hospital and we continued to taper PSL gradually through outpatient visits. Sixty-one days after the administration of PSL, when the drug was reduced to 20 mg/day, we examined him by contrast-enhanced CT. The images showed improvement of abdominal aortic wall thickening (Fig. 2C). We further tapered PSL slowly and discontinued it 141 days after starting. No serious steroid-related adverse events were observed during the course. There was no recurrence of aortitis after the discontinuation of steroids.

**Table 1:** Laboratory data

Complete blood count			Coagulation			Biochemistry		
WBC	14,000	/μL	PT	58	%	TP	6.7	g/dL
Myelo	0.5	%	PT-INR	1.31		T-Bil	2.2	mg/dL
Neu	85	%	APTT	33.2	Sec	D-Bil	0.9	mg/dL
Mono	7.5	%	Fibrinogen	1101	mg/dL	γ-GTP	146	IU/L
Lympho	7	%				AST	66	IU/L
RBC	4.08	× 10^6^/μL				ALT	78	IU/L
Hb	13.2	g/dL				LD	255	IU/L
Hct	39.2	%				BUN	31.7	mg/dL
Plt	32.8	× 10^4^/μL				Cre	1.47	mg/dL
						UA	5.4	mg/dL
						CRP	36.09	mg/dL
						IL-1β	Undetectable	

The recipient’s engraftment was successful without G-CSF administration 15 days after the transplantation. The recipient achieved complete response from sCAEBV.

## Discussion

Although there have been reports of aortitis after the administration of pegfilgrastim in cancer-bearing patients [2, 3], there have been no reports of healthy donors of allogeneic peripheral blood stem cell transplantation. The incidence of aortitis in breast cancer patients treated with pegfilgrastim is reported as 0.3% [2]. Its clinical findings show symptoms such as fever, chest pain, back pain, and muscle pain 13.4 ± 7.6 days after the administration of pegfilgrastim [2]. The clinical course of our case was similar to this description, and since no other cause of aortitis was found, we diagnosed the case as pegfilgrastim-induced vasculitis. In a previously published report, both non-pegylated G-CSF and pegylated G-CSF symptoms appeared in 7–15 days. Although treatment methods vary by report, symptoms subsided for about 7 to 15 days, and it is not clear if there are differences in the timing of onset and duration of symptoms depending by different formulation [4]. In breast cancer patients, there are reports stating that biosimilars among pegylated G-CSF did not cause the disease, but the number of cases differ by report. We must accumulate more cases for further evaluation [2].

Aortitis after the administration of pegfilgrastim is thought to be associated with the activation of neutrophils by G-CSF and excessive secretion of inflammatory cytokines [5]. Interleukin-1β has been reported to be involved in the onset of vasculitis such as Kawasaki disease [6], but the plasma level was below sensitivity in this case (Table 1). In addition, it has been reported that the incidence of drug-induced aortitis differ by ethnicity suggesting a genetic predisposition with people with Asian backgrounds [7].

In this case, the administration of PSL brought rapid improvement of clinical symptoms, laboratory test values, and imaging showing improved aortic wall thickening. This suggests that steroids may be effective in treating pegfilgrastim-induced aortitis in HSCT donors as well as in cancer patients [2, 3]. On the other hand, it is necessary to accumulate more cases and examine the optimal dosage and administration period. When administering G-CSF to healthy donors, the risk of developing not only aortitis but also rare complications such as acute glomerulonephritis and acute lung injury must be taken into consideration because of autoimmune disorders [8, 9].

The use of pegfilgrastim for stem cell harvesting reduces donor hospitalization period. As its use become more common, we suspect the number of similar cases will increase and the possibility of serious adverse events such as aortitis and require treatment may also increase. We must give careful attention to donors’ health management before and after hematopoietic stem cell collection.

## Data Availability

Data sharing not applicable to this article as no datasets were generated or analyzed during the current study.
